# Initial demonstration of the Scratch-PET concept: an intraoperative PET with a hand-held detector

**DOI:** 10.1007/s12194-025-00889-z

**Published:** 2025-03-12

**Authors:** Taiyo Ishikawa, Yuma Iwao, Go Akamatsu, Sodai Takyu, Hideaki Tashima, Takayuki Okamoto, Taiga Yamaya, Hideaki Haneishi

**Affiliations:** 1 Graduate School of Science and Engineering, Chiba University, Chiba, Japan; 2 Institute for Quantum Medical Science, National Institutes for Quantum Science and Technology (QST), Chiba, Japan; 3 Center for Frontier Medical Engineering, Chiba University, Chiba, Japan

**Keywords:** Positron emission tomography (PET), Intraoperative PET imaging, Image-guided surgery, Hand-held detector

## Abstract

Positron emission tomography (PET) is a valuable tool for diagnosing malignant tumors. Intraoperative PET imaging is expected to allow the more accurate localization of tumors that need resections. However, conventional devices feature a large detector ring that obstructs surgical procedures, preventing their intraoperative application. This paper proposes a new PET device, Scratch-PET, for image-guided tumor resection. The key feature of Scratch-PET is its use of a hand-held detector to scan the surgical field, ensuring open space for surgery while measuring annihilation radiation with a fixed detector array placed below the patient. We developed a prototype device using two detectors: the hand-held detector and a fixed detector, to demonstrate the feasibility of the proposed concept. Both detectors consisted of 16 × 16 arrays of lutetium yttrium orthosilicates (3 × 3 × 15 mm^3^) coupled one-to-one with 16 × 16 silicon photomultiplier arrays. The position and orientation of the hand-held detector are tracked using an optical tracking sensor that detects attached markers. We measured a ^22^Na multi-rod phantom and two ^22^Na point sources separately for 180 s while moving the hand-held detector. The rod diameters were 6.0, 5.0, 4.0, 3.0, 2.2, and 1.6 mm. Each point source was placed at the field-of-view center and 35 mm off-center which was outside the sensitive area when the hand-held detector was positioned facing the fixed detector. The 2.2 mm rods were partially resolved, and both point sources were successfully visualized. The potential of the proposed device to visualize small tumors was validated.

**Supplementary Information:**

The online version contains supplementary material available at 10.1007/s12194-025-00889-z.

## Introduction

Surgical tumor resection is one of the primary treatments for many malignant tumors, and complete resection of tumor tissues is crucial for improving curability and postoperative survival rates [1–4]. Tissues where the tumor may have spread are resected, and it is extensive in some cases. On the other hand, unnecessary resection increases the physical burden on patients, and localizing the tumor tissue accurately to minimize the extent of resection is also required.

Various imaging techniques have been studied to provide the location of tumor tissues intraoperatively [5, 6]. Among them, fluorescence imaging has gained significant attention [7–9]. In this approach, fluorescent agents accumulate in the tumor and emit light when excited by external light, enhancing tumor visualization. This technique is characterized by high spatial resolution, making it useful for assessing tumor resection margins. However, due to the significant absorption of fluorescence by biological tissue, the maximum penetration depth is restricted to approximately 5–10 mm [7]. This inherent limitation obstructs the visualization of deep-seated tumors [8].

Nuclear medicine techniques have also been studied [10, 11]. Gamma probes have been proposed [12–21], and some of them aimed to detect sentinel lymph nodes [18, 19, 22]. ^99m^Tc-labeled colloids are used for lymph node visualization, which is followed by pathological diagnosis. In contract, positron emission tomography (PET) radiopharmaceutical, ^18^F-fluorodeoxyglucose (FDG), is often used to detect tumor tissues [23–25]. Positron probes measuring positron directly have been developed [26–29], but they suffer from a short positron range and background noise of annihilation radiation. On the other hand, ^18^F-FDG PET is expected to be able to diagnose malignant tumors as well as metastasis to be resected [30–32]. The annihilation radiations or 511 keV gamma-rays have more penetrating power than fluorescence and positron, enabling us to measure deep-seated tumors.

To use the surgical instruments in the field-of-view (FOV), an open-type PET device, OpenPET, has been proposed, featuring a detector ring shaped like a diagonally cut cylinder [33–35]. Tumor resection on animals guided by OpenPET has been performed, and its utility was demonstrated [36, 37]. A system with a detector mounted on a robotic arm has also been proposed [38]. However, both OpenPET and the robotic arm detector require large equipment, obstructing access of surgical instruments to the FOV. As a relatively compact alternative for intraoperative PET imaging, laparoscopic-type systems have also been proposed [39–41]. However, the diameter of the laparoscopic device must be smaller than the inner diameter of the laparoscopic trocar, which severely limits the size of the detector. This results in constraints on the FOV and detector sensitivity. Furthermore, because the device is inserted into the body, radioactivity accumulated outside the FOV in the body increases random coincidences, degrading the image quality.

In this study, we propose a novel concept for intraoperative PET imaging by scanning the surgical field with a hand-held detector. We named this concept “Scratch-PET” in analogy to scratch cards. The key feature of Scratch-PET is its use of a hand-held detector in place of a detector ring. Annihilation radiation will be measured with the hand-held detector and a fixed detector array placed below the patient. This compact design will enable the imaging of a wide area while ensuring an open space for surgery. Here, we report on the development of a prototype device and the first imaging demonstration. We experimentally verified the feasibility of PET imaging with moving the hand-held detector.

## Methods

First, we introduce the concept of the proposed Scratch-PET. Second, we explain our prototype device, and third, we present an image reconstruction method dedicated to Scratch-PET.

### Proposed Scratch-PET concept

**Fig. 1:**
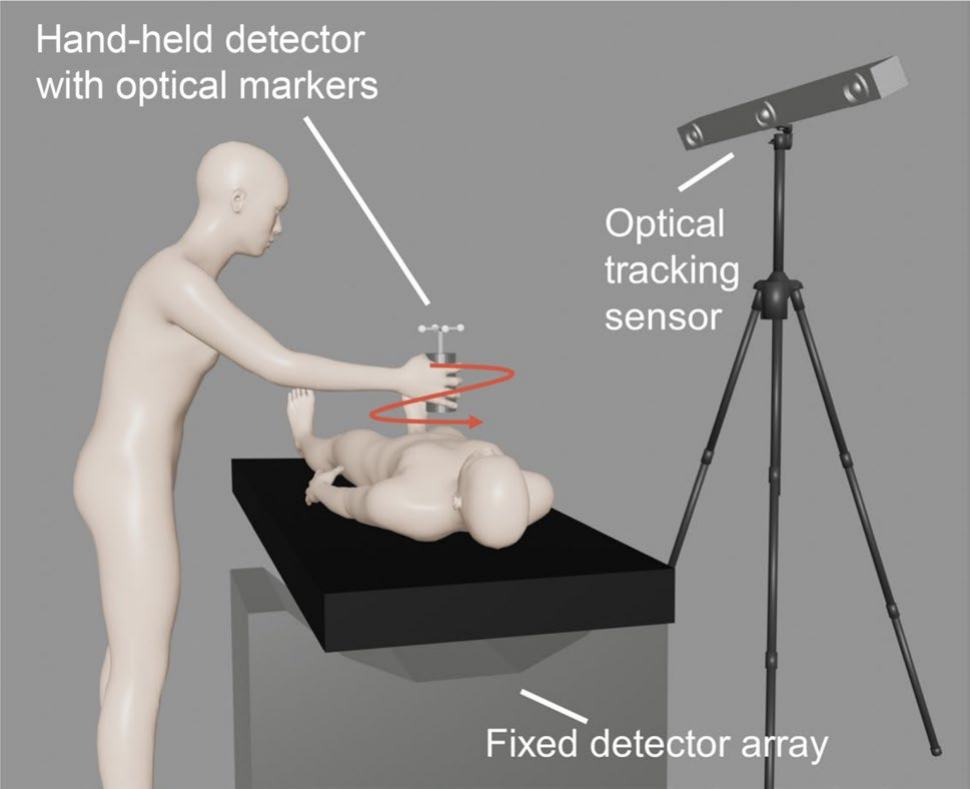
Schematic illustration of Scratch-PET. Annihilation radiation is measured between a hand-held detector and a fixed detector array. The operator manually scans the surgical field with the hand-held detector. The positions of the optical markers, which are attached to the hand-held detector, are measured by an optical tracking sensor to obtain the positional information of the hand-held detector

Figure 1 illustrates our proposed Scratch-PET. The operator scans the surgical field with the hand-held detector, measuring annihilation radiation in conjunction with the fixed detector array below the patient. The positions of optical markers attached at the top of the hand-held detector are measured by an optical tracking sensor. The position and orientation of the hand-held detector are computed from the position of the optical markers. The measurement time synchronizes the detector position and the coincidence data. Scratch-PET reconstructs the acquired data immediately and presents 3D PET images of the scanned region to the operator.

### Prototype device

**Fig. 2:**
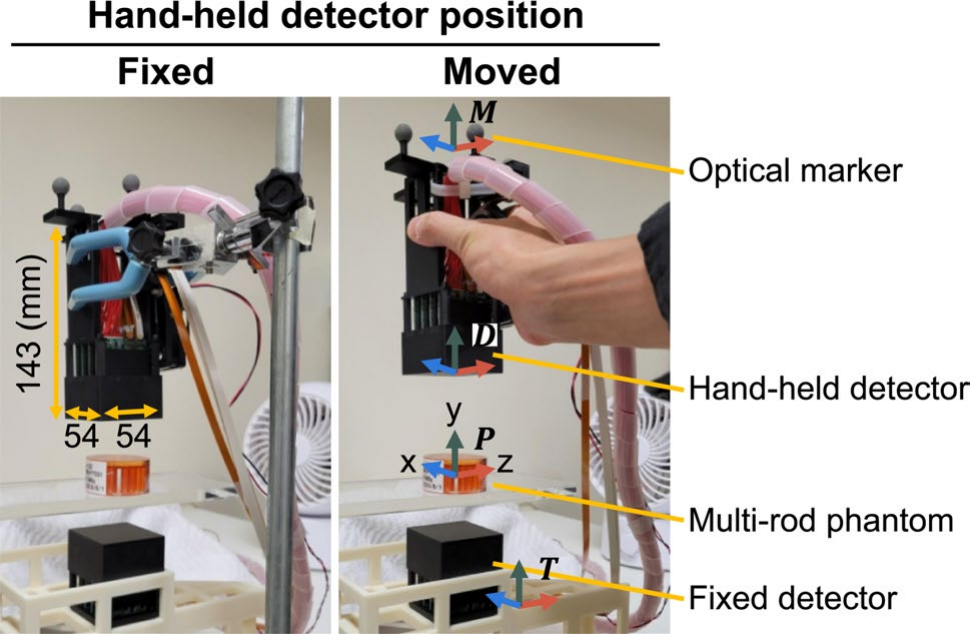
Setup for multi-rod phantom imaging using the prototype device. We compared the measurements when the hand-held detector was moved manually and when the hand-held detector was positioned facing the fixed detector while measuring the annihilation radiation. The *x*-, *y*-, and *z*-axes of marker coordinates ${\varvec{M}}$, hand-held detector coordinates ${\varvec{D}}$, PET image coordinates ${\varvec{P}}$, and tracking sensor coordinates ${\varvec{T}}$ were indicated by blue, green, red arrow, respectively

The prototype device consisted of the PET detectors, the position tracking system, and the workstation, which was used for data acquisition and image reconstruction. We used two detectors: a hand-held detector and a fixed detector (Fig. 2). We used time-of-flight (TOF)-PET detector modules (C13500 series, Hamamatsu Photonics K. K., Hamamatsu, Japan) [42]; the details are described in Ref. [42]. Each detector had a scintillator array that consisted of a 16 × 16 array of lutetium yttrium orthosilicate (LYSO) crystals (3 × 3 × 15 mm^3^) and had a sensitive area of 51 × 51 mm^2^. The scintillator array was coupled one-to-one to a 16 × 16 array of silicon photomultiplier (SiPM). The size of the hand-held detector was 54 × 54 × 143 mm^3^, and the weight was 466 g. The energy window of the detectors was set to 400–600 keV. Air circulators were used to cool the detectors. The single events were registered in the workstation via an optical fiber cable. Software-based coincidence detection was conducted every 5 s by comparing the detection time of the list-mode data. The coincidence timing window was 1.8 ns. A delayed coincidence method was implemented for the random correction. The delayed coincidence timing window was also 1.8 ns with a delay time of 30 ns. The 3D PET images were reconstructed from the coincidence data and displayed on the monitor near the prototype device.

To track the motion of the hand-held detector, we used OptiTrack v120: Trio (Natural Point Inc., Corvallis, OR, USA). Four optical markers were attached to the hand-held detector via a cross-shaped component (Fig. 2). The positions of the markers were measured with the optical tracking sensor on the tracking sensor coordinates ${\varvec{T}}$. The tracking sensor coordinates ${\varvec{T}}$ were determined by placing calibration markers. The marker coordinates ${\varvec{M}}$ were defined at the center of gravity of the optical markers. The hand-held detector coordinates ${\varvec{D}}$ were defined at the center of the hand-held detector’s surface. The PET image coordinates ${\varvec{P}}$ was set at the FOV center. The position and orientation of the coordinate systems are shown in Fig. 2. The coordinates transformation was represented as a 4 × 4 homogeneous transformation matrix

1 $$\begin{array}{c}\varvec{H}=\left[\begin{array}{cccc}& {\varvec{R}}& & {\varvec{t}}\\ 0& 0& 0& 1\end{array}\right]\end{array}$$

where ${\varvec{R}}$ was a 3 × 3 rotation matrix and ${\varvec{t}}$ was a 3 × 1 translation vector. The transformation from the PET image coordinates to the hand-held detector coordinates was defined using product of the homogeneous transformation matrices as

2 $$\begin{array}{c}{{\varvec{H}}}_{{\varvec{P}}{\varvec{D}}}={{\varvec{H}}}_{{\varvec{P}}{\varvec{T}}}{{\varvec{H}}}_{{\varvec{T}}{\varvec{M}}}{{\varvec{H}}}_{{\varvec{M}}{\varvec{D}}}\end{array}$$

where ${{\varvec{H}}}_{{\varvec{P}}{\varvec{D}}}$, ${{\varvec{H}}}_{{\varvec{P}}{\varvec{T}}}$, ${{\varvec{H}}}_{{\varvec{T}}{\varvec{M}}}$**,** and ${{\varvec{H}}}_{{\varvec{M}}{\varvec{D}}}$ were the homogenous transformation matrix from the PET image coordinates to the hand-held detector coordinates, from the PET image coordinates to the tracking sensor coordinates, from the tracking sensor coordinates to the marker coordinates, and from the marker coordinates to the hand-held detector coordinates, respectively. The relative position between the fixed detector and the calibration markers determined ${{\varvec{H}}}_{{\varvec{P}}{\varvec{T}}}$. The measurement data with the optical tracking sensor determined ${{\varvec{H}}}_{{\varvec{T}}{\varvec{M}}}$. ${{\varvec{H}}}_{{\varvec{M}}{\varvec{D}}}$ was determined based on the design plan.

To obtain accurate energy information about the annihilation radiation, the non-linearity of the output signal for the radiation energy was corrected. We measured the radiation from a ^22^Na point source and the background radiation from ^176^Lu in the LYSO crystals for energy calibration [43]. The energy resolution for the 511 keV radiation was calculated from the corrected data of the ^22^Na radiation measurement and expressed by full width at half maximum (FWHM).

To estimate an accurate detection time, the variation of the time stamp among SiPM channels was corrected. We measured the radiation from a ^68^Ge line source (270 mm long) with the hand-held detector and the fixed detector positioned opposite each other at 130 mm. This line source was positioned at the middle of the detectors. To simulate a flat plate source, the line source was moved back and forth for 90 mm in a direction parallel to the detector surfaces. The time stamp correction factor for each channel was estimated using the iterative method [44]. The correction factor was updated by adding half of the peak position offset in the timing spectra. Coincidence detection was conducted on the single data with the corrected time stamp to calculate the TOF resolution.

### Image reconstruction method for Scratch-PET

The list-mode TOF 3D dynamic row-action maximum likelihood algorithm (LM-TOF-3D-DRAMA) [45, 46] was used for the image reconstruction. We added the functionality to accommodate changes in the detector position to the LM-TOF-3D-DRAMA by introducing the motion correction technique [47–49]. The position of the crystals on the hand-held detector side was computed from the position of the detector measured by the optical tracking sensor. The TOF kernel and detector response were modeled by the Gaussian function [50]. In LM-TOF-3D-DRAMA, the iterations of the projection–backprojection computation update the reconstructed image. This computation was implemented on a graphics processing unit of GeForce RTX 3090 with 24 GB memory (NVIDIA Corporation, Santa Clara, CA, USA).

Detection sensitivity in the FOV depends on the detector geometry. Thus, the global sensitivity image (GSI) should be computed for each detector position. Although on-the-fly computation of GSIs is necessary for quick reconstruction, the computational cost is an issue. Here, we downsized the image to reduce the computational cost. Trilinear interpolation recovered the original resolution of the degraded GSIs.

**Fig. 3:**
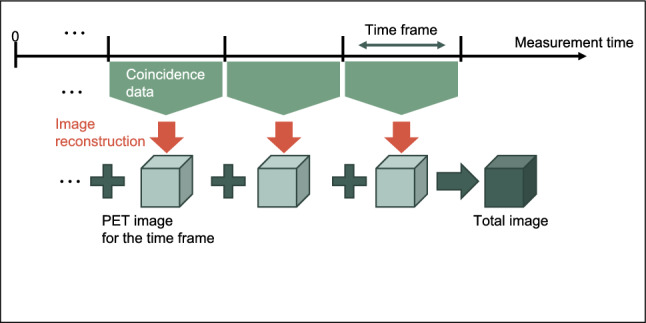
Schematic illustration of the proposed image reconstruction. The reconstruction process was performed for each time frame. All the images were added to output the total image

The computational cost of the list-mode image reconstruction is proportional to the number of coincidence data. Thus, we divided the coincidence data into time frames (Fig. 3). Referring to a motion correction algorithm using multiple acquisition frames [51], we reconstructed the frame images only from the added coincidence data after the data acquisition at regular intervals. These frame images were added to output a total image from the start of the data acquisition.

### Experiments

**Fig. 4:**
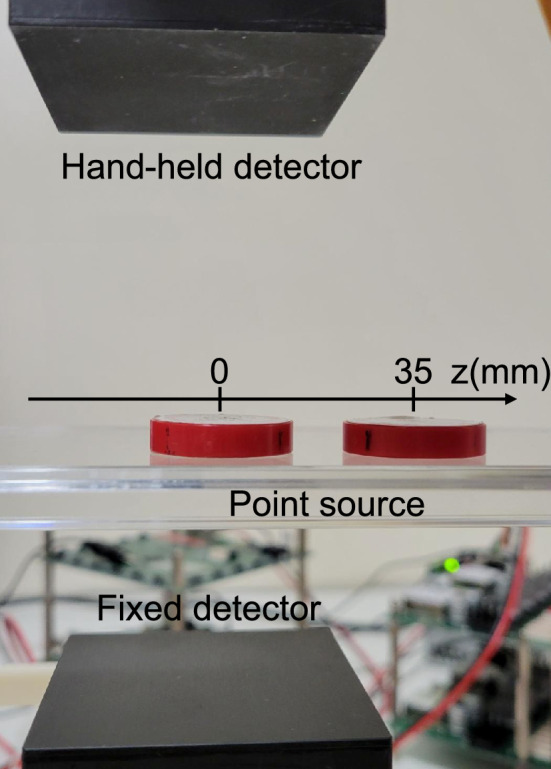
The point source was placed at the FOV center and 35 mm off-center in the horizontal direction which is outside the sensitive area when the hand-held detector was positioned facing the fixed detector

A ^22^Na-filled multi-rod phantom [52] (Fig. 2) and two ^22^Na point sources (Fig. 4) were measured separately. The rod diameters of the multi-rod phantom were 6.0, 5.0, 4.0, 3.0, 2.2, and 1.6 mm. The length of each rod was 15 mm. The activity of the multi-rod phantom was 0.19 MBq. The FOV center was set at 50 mm above the fixed detector. Each point source was placed at the FOV center and 35 mm off-center in the horizontal direction which was outside the sensitive area when the hand-held detector was positioned facing the fixed detector, respectively (Fig. 4). The activity of each point source was 0.21 MBq and 0.36 MBq, respectively.

Two different hand-held detector geometries were compared for each measurement: a manual movement and a fixed geometry (Fig. 2). In the fixed geometry, the hand-held detector and the fixed detector were positioned face-to-face at a distance of 115 mm.

We used the same device setup in all measurements. The optical tracking sensor was positioned 1 m above the fixed detector, oriented to ensure that both detectors were within the sensitive area of the tracking sensor, and the position of the hand-held detector was measured at 20 frames per second. The data acquisition time was 180 s. The interval of the image reconstructions was 10 s. The image reconstructions were performed for a 100 × 100 × 100 mm^3^ FOV with a voxel size of 1.0 × 1.0 × 1.0 mm^3^. For each reconstruction process, the list-mode data were divided into four subsets and used for the DRAMA update. The number of pixels on each axis of the generated GSI was reduced by two-thirds. Before starting the data acquisition, the hand-held detector was positioned such that the phantoms were outside the sensitive area. Consequently, the events measured before the phantom entered the sensitive area of the hand-held detector were regarded as noise. To prevent the reconstruction of such noise, the acquisition of over 3000 events triggered the reconstruction process. As a figure of merit for TOF information, we also reconstructed the measurement data of the multi-rod phantom without TOF information.

## Results

The energy resolution for the 511 keV radiation was 13.5% FWHM for the hand-held detector and 14.7% FWHM for the fixed detector. The TOF resolution of the detector pair was improved from 1002 ps FWHM to 348 ps FWHM by the timing calibration.

**Fig. 5:**
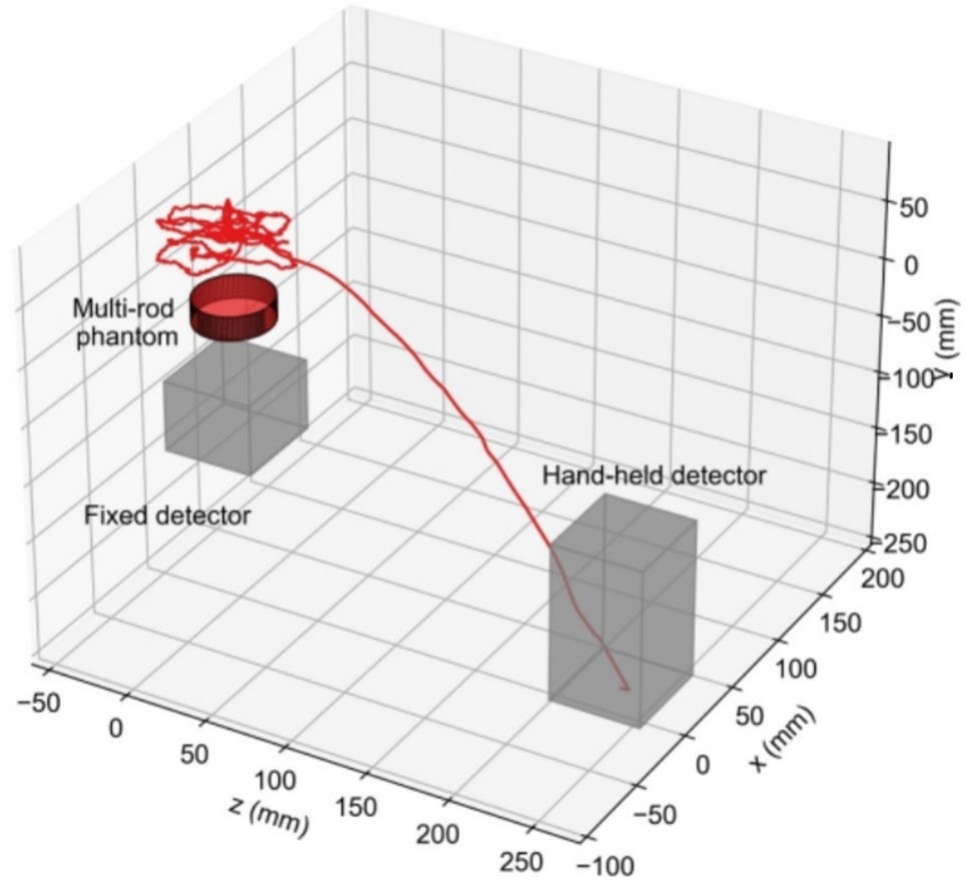
Scanning trajectory of the center of the hand-held detector’s surface in the PET image coordinates for the multi-rod phantom imaging. The *x* and *z* directions were the horizontal direction, and the y direction was the vertical direction. The arrangement of the phantom, the fixed detector, and the hand-held detector at the initial position is shown. The detector was moved randomly

**Fig. 6:**
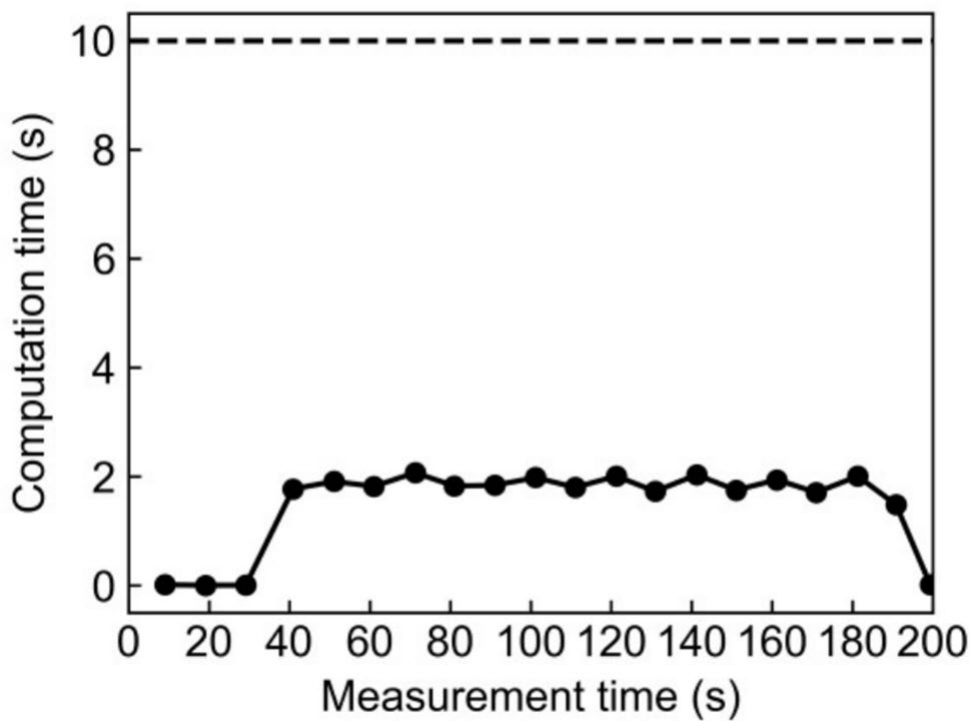
Computational time for each reconstruction process in the multi-rod phantom imaging with moving the hand-held detector. The horizontal axis is the measurement time when each image reconstruction process was finished. The computation times (solid line) were shorter than the reconstruction interval of 10 s (dashed line) in all the reconstruction processes

Supplementary material 1 shows a demonstration video of the multi-rod phantom imaging with moving the hand-held detector. The hand-held detector was moved manually, resulting in an irregular scanning trajectory (Fig. 5). The position and the orientation of the hand-held detector were measured continuously with the optical tracking sensor without interruption. The reconstruction process began 24 s after the hand-held detector was lifted. The computational time for the reconstruction processes was less than 2 s, which was shorter than the reconstruction interval of 10 s. This fast computation enabled the constant image display (Fig. 6).

**Fig. 7:**
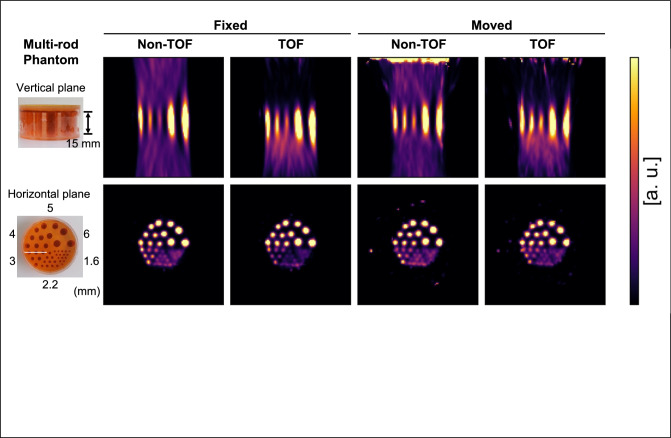
The ^22^Na multi-rod phantom and the 3D reconstructed images after 180 s measurement. The reconstructed images of when the hand-held detector was moved or fixed, and whether TOF information was used or not used were compared. All rods greater than 3 mm in diameter were resolved, and the rods with 2.2 mm in diameter were partially resolved in all conditions

Figure 7 shows the reconstructed images of the multi-rod phantom with the fixed and moved hand-held detector. The random fraction was estimated by dividing the number of the delayed coincidence events by the number of the prompt coincidence events. The random fractions were 0.26% and 0.31% when the hand-held detector was fixed and moved, respectively. All the rods with a diameter greater than 3.0 mm were resolved and the rods with a diameter greater than 2.2 mm were partially resolved in all conditions. The peak-to-valley ratio of the line profiles on the 3 mm rods (the white line shown in the bottom left image in Fig. 7) were 5.0 and 5.8 for non-TOF and TOF with fixing the detector and 3.9 and 4.0 for non-TOF and TOF with moving the detector, respectively. The streak artifacts in the vertical planes were reduced using the TOF information.

**Fig. 8:**
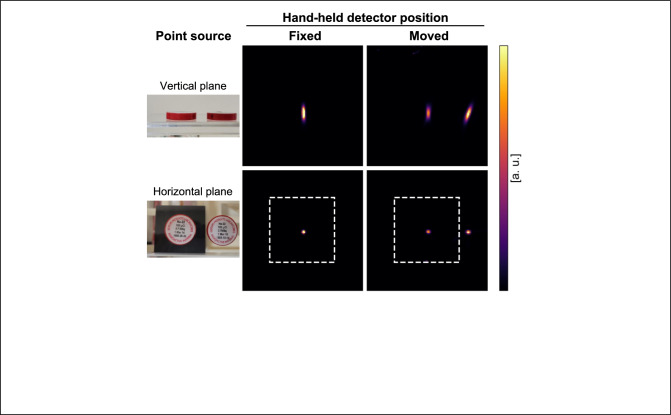
The two ^22^Na point sources and the 3D reconstructed images after 180 s measurement. The position of the fixed detector in horizontal plane is shown using white rectangle. The point source at 35 mm off-center was visualized when the hand-held detector was moved, while the point source was not visualized when the hand-held detector was positioned facing the fixed detector

Figure 8 shows the reconstructed images of the two point sources with the fixed and moved hand-held detector. The position of the fixed detector in horizontal plane was shown using a white rectangle. The random fractions were 0.23% and 0.51% when the hand-held detector was fixed and moved, respectively. The point source at the FOV center was visualized at the same position in both conditions, confirming that the coordinate transformation was performed correctly. The point source at 35 mm off-center was visualized when the hand-held detector was moved, whereas it was not visualized when the hand-held detector was fixed.

Table 1 shows the spatial resolution of the point source. At the FOV center, the spatial resolution when the hand-held detector was moved was worse than that when the hand-held detector was fixed. The spatial resolution at the 35 mm off-center was better in the y direction (vertical direction) than at the FOV center when the hand-held detector was moved. On the other hand, the point source at the 35 mm off-center was not visualized when the hand-held detector was fixed because the source position was outside the sensitive area of the detectors.

**Table 1:** The spatial resolution of the point source. The spatial resolution at the FOV center, when the hand-held detector was moved, was worse than that when the hand-held detector was fixed. The spatial resolution at the 35 mm off-center was better in the y direction (vertical direction) than at the FOV center when the hand-held detector was moved. In contrast, the point source was not visualized when the hand-held detector was fixed because the source position was outside the sensitive area of the detectors

Point source position	Hand-held detector position	FWHM *x* (mm)	FWHM *y* (mm)	FWHM *z* (mm)
FOV center	Moved	2.2	7.7	2.5
	Fixed	2.0	7.3	1.9
35 mm off-center	Moved	2.1	5.5	2.5
	Fixed	NA	NA	NA

## Discussion

In this study, we demonstrated PET imaging with moving the hand-held detector which is intended to support surgical guidance. Although the prototype device was small, its spatial resolution in the horizontal plane was better than that of clinical whole-body PET systems [53] (Table 1). This result supports the potential of the prototype device to visualize small tumors that appear in whole-body PET images intraoperatively. The spatial resolution at the FOV center was worse when the hand-held detector was moved than when it was fixed because the sampling frequency of the tracking sensor limited the accuracy of the positional measurement of the detector (Fig. 7, Table 1). High sampling frequency of the optical tracking sensor is expected to improve the spatial resolution. It should be noted that the computational cost of GSI is increased according to the sampling frequency of the tracking sensor.

Moving the hand-held detector enabled to visualize the point source outside the sensitive area when the hand-held detector was fixed (Fig. 8, Table 1). We showed that the sensitive area was expanded by moving the detector.

The proposed image reconstruction method enabled us to obtain reconstructed images with a shorter delay than 2 s. Although a previous study mentioned that computational cost could be a bottleneck for a quick reconstruction process [38], our method addressed this problem. A limitation of our reconstruction method is that it only computes the geometrical factor of the system sensitivity for GSI. Normalization should be applied to enhance the uniformity of the system sensitivity across the FOV [54]. In future work, we should develop normalization methods for moving detectors.

The artifacts in the reconstructed images in the multi-rod phantom imaging with moving the hand-held detector were likely to be caused by the LORs in low-sensitivity areas. These LORs were emphasized by the geometrical sensitivity correction (Fig. 7). The artifacts at the top of the vertical planes were because the hand-held detector was entered into the reconstruction FOV, causing the sensitivity correction error. Streak artifacts along the vertical direction were observed in all the reconstructed images (Figs. 7, 8). The streak artifacts are an inherent problem for limited-angle PET systems, such as dual-head systems for positron emission mammography [55–59]. Extensive scanning of the hand-held detector expanding the sensitive area for greater angular coverage [60] and modeling the point spread function deformation [61, 62] can help reduce the streak artifacts. Improving the TOF resolution of the detector [58, 60] is also an effective strategy. We showed that the streak artifacts were reduced even with 348 ps TOF resolution, corresponding to 52 mm spatial resolution (Fig. 7). Recently, a detector pair with 30 ps TOF resolution [63], which corresponds to 4.5 mm spatial resolution, demonstrated to obtain cross-sectional images without detector rotation [64]. Such ultrafast TOF detectors are expected to eliminate streak artifacts.

Only a simple phantom was measured in this study because we focused on demonstrating PET imaging with moving the hand-held detector. In actual scenarios, the contrast between the tumors and background activity should be considered. Attenuation and scattering of the annihilation radiation will likely increase compared to the setup of this study. Furthermore, the intensity of radioactivity depends on the injection timing. For example, in the previous study [65], ^18^F-FDG was administrated approximately 3 h before surgery. The radioactivity decays to about 32% of the initial dose. The Scratch-PET has a disadvantage in system sensitivity compared with a full-ring PET, and may not provide sufficient image quality for tumor detection. To investigate the tumor detectability, we performed a preliminary imaging simulation with a contrast phantom (Appendix 1). We modeled a Scratch-PET device using five detectors: a hand-held detector and a 2 × 2 fixed detector array, to increase the system sensitivity and expand the sensitive area. The contrast phantom was composed of four spherical sources mimicking tumors and a cylindrical background. The phantom was filled with water, and positrons without kinetic energy were generated directly to approximate the decay process of positron sources. The spherical sources had 4.0, 6.0, 8.0, and 10.0 mm in diameter. The activity intensity of the background was set at 1.6 kBq/ml, and the spherical sources had an eight times higher activity than the background. As a result, the spherical sources greater than 6.0 mm in diameter were visualized. This result showed the potential of Scratch-PET to visualize tumors. In future work, we will investigate the tumor imaging performance with a human phantom in a similar condition to the actual surgery.

Scratch-PET would be useful not only for intraoperative tumor imaging, but also for biopsy guidance. Gamma probe-guided lymph node biopsy using ^18^F-FDG and ^89^Zr-nanocolloidal albumin were reported [66, 67]. Scratch-PET may be able to provide the positional information of the target lymph node with better spatial resolution than the gamma probes.

## Conclusion

We proposed the Scratch-PET concept for intraoperative PET imaging. Our first prototype device featuring manual movement of its hand-held detector successfully demonstrated to obtain 3D PET images with 2.2 mm rod resolution. The sensitive area was expanded when moving the hand-held detector compared with the case when placing the hand-held detector facing the fixed detector. Additionally, the image reconstruction with a shorter delay than 2 s allowed the operator to check the reconstructed images during measurement. We showed the potential of the proposed concept to visualize the small tumors with wide FOV.

## Electronic supplementary material

Below is the link to the electronic supplementary material.

Supplementary file1 (DOCX 34 KB)

Supplementary file2 (MP4 215822 KB)

## Figures and Tables

**Fig. 9:**
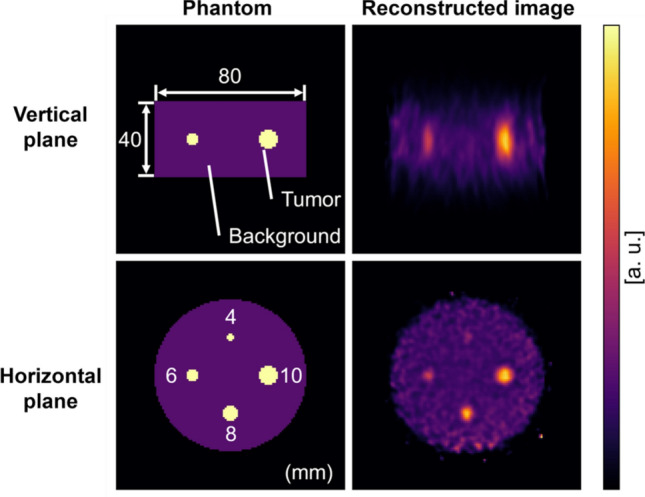
The activity distribution and reconstructed image of the cylindrical contrast phantom. The spherical sources over 6 mm were visualized

## Data Availability

The data of this study are available from the corresponding author upon reasonable request.

## Appendix 1: Imaging simulation of a contrast phantom

We investigated the tumor detection ability of Scratch-PET using Geant4 Monte Carlo simulation. We modeled a Scratch-PET device using five detectors: a hand-held detector and a 2 × 2 fixed detector array. all detectors consist of 16 × 16 arrays of lutetium yttrium orthosilicates (3 × 3 × 15 mm^3^). We assumed the crystal array was coupled one-to-one with 16 × 16 arrays of silicon photomultipliers. TOF resolution was set at 348 ps, which was the same as the prototype device. An irregular trajectory was configured as the hand-held detector movement. For simplicity, the detection surface of the hand-held detector was kept pointing to the negative y-axis (vertically downward) direction. We assumed that the position of the hand-held detector was precisely measured by an external sensor.

A cylindrical contrast phantom that mimics the radioactive concentration in tumors was used as an imaging object. Positrons without kinetic energy were generated directly to approximate the decay process of positron sources. The phantom was 80 mm in diameter and 40 mm in height and consisted of water with background activity. The background activity concentration was set at 1.6 kBq/ml (corresponding to a typical clinical ^18^F-fluorodeoxyglucose injection of 4.5 MBq/kg and 3 h for decay [65]). Four spherical sources were embedded in the cylindrical background. The diameters of the spherical sources were 4.0, 6.0, 8.0, and 10.0 mm. The activity in the spherical sources was eight times that of the background one. The data acquisition time was 120 s.

Figure 9 shows the phantom and reconstructed image. The spherical sources over 6 mm in diameter were visualized. This result suggested the possibility of visualizing the tumor with the expected radioactivity intensity.
