# Supplementary material for: Initial demonstration of the Scratch-PET concept: an intraoperative PET with a hand-held detector
Source: Radiol Phys Technol. 2025 Mar 12;18(2):407–16. doi: 10.1007/s12194-025-00889-z (PMC12103487; doi:10.1007/s12194-025-00889-z)
Supplement: Supplementary file 1 — Supplementary file1 (DOCX 34 KB) [file 12194_2025_889_MOESM1_ESM.docx]

**Initial demonstration of the Scratch-PET concept:
an intraoperative PET with a hand-held detector**

**Radiological Physics and Technology**

**Taiyo Ishikawa^1,2^, Yuma Iwao^2^, Go Akamatsu^2^, Sodai Takyu^2^, Hideaki Tashima^2^, Takayuki Okamoto^3^, Taiga Yamaya^2,3^ and Hideaki Haneishi^3^**

^1^ Graduate School of Science and Engineering, Chiba University, Chiba, Japan

^2^ Institute for Quantum Medical Science, National Institutes for Quantum Science and Technology (QST), Chiba, Japan

^3^ Center for Frontier Medical Engineering, Chiba University, Chiba, Japan

Corresponding author: Taiyo Ishikawa (t_ishikawa@chiba-u.jp)

**Supplementary material 1**

The movie began when the data acquisition of the annihilation radiation started. The hand-held detector was lifted 24 s after starting the radiation measurement. The reconstruction process started at 45 s, when the amount of projection data exceeded the threshold of 3000 counts. Then, the first reconstructed image was displayed at 47 s. The reconstructed images were updated on the monitor during 150 s.
